# Supplementary material for: The Influence of the pH and Salinity of Water in Breeding Sites on the Occurrence and Community Composition of Immature Mosquitoes in the Green Belt of the City of São Paulo, Brazil
Source: Insects. 2021 Sep 5;12(9):797. doi: 10.3390/insects12090797 (PMC8469630; doi:10.3390/insects12090797)
Supplement: Supplementary file 1 [file insects-12-00797-s001.zip › insects-1312934-supplementary.pdf]

Supplementary information

Table S1. Number of mosquito species collected by breeding site in two remnants of Atlantic Forest in the city of São Paulo, Brazil.

**Area**	**Species**	**Artificial**	**Epiphytic bromeliads**	**Ground bromeliads**	**Bamboo hole**	**Lake**	**Tree hollow**	**Bamboo hollow**	**Rock**	**Ponds**	**Total**
**Cantareira State Park**	*Ad. (Ady.) squamipennis*	0	0	0	0	0	0	0	-	0	0
	Ae. (Grg.) fluviatilis	0	0	0	0	0	0	0	-	0	0
	Ae. (Och.) crinifer	0	0	0	0	0	0	0	-	20	20
	Ae. (Och.) nubilus	0	0	0	0	0	0	0	-	0	0
	Ae. (Och.) rhyacophilus	0	0	0	0	0	0	0	-	0	0
	Ae. (Och.) scapularis	0	0	0	0	0	0	0	-	0	0
	Ae. (Pro.) terrens	0	0	0	0	0	0	0	-	0	0
	Ae. (Stg.) aegypti	35	0	0	0	0	0	22	-	0	57
	Ae. (Stg.) albopictus	31	4	0	0	0	0	7	-	0	42
	An. (Ker.) bellator	**0**	**1**	**0**	**0**	**0**	**0**	**0**	-	**0**	**1**
	An. (Ker.) cruzii	0	253	4	0	0	0	0	-	0	257
	An. (Nys.) strodei	4	0	0	0	10	0	0	-	0	14
	Cx. (Car.) iridescens	493	18	0	276	0	5	102	-	10	904
	Cx. (Cux.) brami	0	0	0	0	0	0	0	-	87	87
	Cx. (Cux.) chidesteri	0	0	0	0	0	0	0	-	0	0
	Cx. (Cux.) coronator	16	0	0	0	0	0	0	-	0	16
	Cx. (Cux.) dolosus	151	7	0	0	4	67	0	-	347	576
	Cx. (Cux.) eduardoi	45	0	0	0	0	6	0	-	100	151
	Cx. (Cux.) lygrus	0	0	0	0	0	0	0	-	36	36
	Cx. (Cux.) mollis	0	0	0	0	0	0	0	-	2	2
	Cx. (Cux.) nigripalpus	0	0	0	0	0	0	0	-	2	2
	Cx. (Cux.) quinquefasciatus	0	0	0	0	0	0	0	-	0	0
	Cx. (Cux.) restuans	**0**	**0**	**0**	**0**	**0**	**0**	**0**	-	**3**	**3**

Cx. (Mcx.) albipes	4	135	2	0	0	0	0	-	0	141
Cx. (Mcx.) aphylactus	0	0	0	0	0	0	0	-	0	0
Cx. (Mcx.) aureus	0	0	0	0	0	0	0	-	0	0
Cx. (Mcx.) daumasturus	0	1	0	0	0	0	0	-	0	1
Cx. (Mcx.) dubitans	0	19	0	0	0	0	0	-	0	19
Cx. (Mcx.) fuscatus	0	5	0	0	0	0	0	-	0	5
Cx. (Mcx.) imitator	3	297	10	2	0	0	0	-	0	312
Cx. (Mcx.) inimitabilis	0	6	0	0	0	0	0	-	0	6
Cx. (Mcx.) lanei	0	32	0	0	0	0	0	-	0	32
Cx. (Mcx.) neglectus	0	14	0	0	0	0	0	-	0	14
Cx. (Mcx.) pleuristriatus	0	119	109	0	0	0	0	-	0	228
Cx. (Mcx.) reducens	**0**	**5**	**0**	**0**	**0**	**0**	**0**	-	**0**	**5**
Cx. (Mcx.) worontzowi	0	151	0	0	0	0	0	-	0	151
Cx. (Mel.) akritos	0	0	0	0	0	0	0	-	0	0
Cx. (Mel.) bahiensis	0	0	0	0	66	0	0	-	34	100
Cx. (Mel.) intrincatus	4	3	0	0	23	0	0	-	5	35
Cx. (Mel.) pilosus	0	0	0	0	0	0	0	-	0	0
Cx. (Mel.) ribeirensis	0	0	0	0	0	0	0	-	0	0
Cx. (Mel.) vaxus	0	0	0	0	212	0	0	-	0	212
Cx. ocellatus	0	43	0	8	0	0	0	-	0	51
Hg. (Con.) leucocelaenus	**20**	**0**	**0**	**5**	**0**	**37**	**9**	-	**0**	**71**
Li. durhami	877	0	0	0	0	0	0	-	0	877
Lu. (Lut.) bigoti	3	0	0	0	0	1	0	-	23	27
Ma. (Man.) indubitans	0	0	0	0	0	0	0	-	0	0
Ps. (Jan.) albigenu	0	0	0	0	0	0	0	-	0	0
Ps. (Jan.) ferox	0	0	0	0	0	0	0	-	3	3
Ru. (Run.) cerqueirai	**0**	**0**	**0**	**7**	**0**	**0**	**0**	-	**0**	**7**
Sa. (Sab.) purpureus	0	0	0	0	0	3	0	-	0	3

	Sh. fluviatilis	**0**	**0**	**0**	**99**	**0**	**0**	**46**	-	**0**	**145**
	Tr. pallidiventer	**2**	**0**	**0**	**23**	**0**	**0**	**3**	-	**0**	**28**
	Tx. (Lyn.) portoricensis	0	0	0	0	0	0	0	-	0	0
	Tx. (Lyn.) theobaldi	3	0	0	0	0	0	0	-	0	3
	Tx. tricophygus	**0**	**3**	**0**	**0**	**0**	**0**	**0**	-	**0**	**3**
	Ur. (Ura.) geometrica	0	0	0	0	1	0	0	-	0	1
	Ur. (Ura.) lowii	0	0	0	0	1	0	0	-	0	1
	Ur. (Ura.) pulcherrima	0	0	0	0	0	0	0	-	0	0
	Wy. (Mim.) oblita	**0**	**0**	**0**	**5**	**0**	**0**	**0**	-	**0**	**5**
	Wy. (Pho.) davisi	0	117	4	0	0	0	0	-	0	121
	Wy. (Pho.) edwardsi	0	24	0	0	0	0	0	-	0	24
	Wy. (Pho.) incaudata	0	0	0	0	0	0	0	-	0	0
	Wy. (Pho.) pallidoventer	0	58	3	0	0	0	0	-	0	61
	Wy. (Pho.) palmata	0	3	0	0	0	0	0	-	0	3
	Wy. (Pho.) pilicauda	0	0	0	0	0	0	0	-	0	0
	Wy. (Pho.) splendida	0	0	0	0	0	0	0	-	0	0
	Wy. (Pho.) theobaldi	3	192	9	0	0	0	0	-	0	204
	Wy. (Spi.) airosai	**0**	**1**	**0**	**0**	**0**	**0**	**0**	-	**0**	**1**
	Wy. (Wyo.) lutzi	**0**	**0**	**0**	**9**	**0**	**0**	**0**	-	**0**	**9**
	Wy. aporonoma	0	0	0	0	0	0	0	-	0	0
	Wy. personata	**0**	**0**	**0**	**4**	**0**	**0**	**0**	-	**0**	**4**
	Wy. serratoria	0	0	0	0	0	0	0	-	0	0
	Total	1694	1511	141	438	317	119	189	0	672	5081
Capivari-Monos conservation area	***Ad. (Ady.) squamipennis***	**0**	**0**	**0**	**-**	**1**	-	**0**	**0**	**0**	**1**
	Ae. (Grg.) fluviatilis	**229**	**0**	**0**	**-**	**5**	-	**0**	**9**	**0**	**243**
	Ae. (Och.) crinifer	6	0	0	-	47	-	0	0	73	126
	Ae. (Och.) nubilus	**0**	**0**	**0**	**-**	**0**	-	**0**	**0**	**6**	**6**
	Ae. (Och.) rhyacophilus	**42**	**0**	**0**	**-**	**0**	-	**0**	**145**	**0**	**187**

Ae. (Och.) scapularis	**20**	**0**	**0**	**-**	**5**	-	**0**	**12**	**37**	**74**
Ae. (Pro.) terrens	**0**	**0**	**0**	**-**	**0**	-	**3**	**0**	**0**	**3**
Ae. (Stg.) aegypti	12	0	0	-	0	-	0	0	0	12
Ae. (Stg.) albopictus	73	2	0	-	0	-	26	0	0	101
An. (Ker.) bellator	0	0	0	-	0	-	0	0	0	0
An. (Ker.) cruzii	0	108	12	-	0	-	0	0	0	120
An. (Nys.) strodei	0	0	0	-	183	-	0	0	12	195
Cx. (Car.) iridescens	37	0	0	-	0	-	31	0	0	68
Cx. (Cux.) brami	7	0	0	-	6	-	0	0	13	26
Cx. (Cux.) chidesteri	**0**	**0**	**0**	**-**	**3**	-	**0**	**0**	**0**	**3**
Cx. (Cux.) coronator	104	0	0	-	7	-	0	8	111	230
Cx. (Cux.) dolosus	307	0	0	-	53	-	0	77	7	444
Cx. (Cux.) eduardoi	75	0	3	-	40	-	0	3	9	130
Cx. (Cux.) lygrus	0	0	0	-	4	-	0	0	2	6
Cx. (Cux.) mollis	15	0	0	-	7	-	0	9	0	31
Cx. (Cux.) nigripalpus	0	0	0	-	0	-	0	0	6	**6**
Cx. (Cux.) quinquefasciatus	**171**	**4**	**0**	**-**	**0**	-	**0**	**14**	**9**	**198**
Cx. (Cux.) restuans	0	0	0	-	0	-	0	0	0	0
Cx. (Mcx.) albipes	8	89	35	-	0	-	18	0	0	150
Cx. (Mcx.) aphylactus	**0**	**0**	**3**	**-**	**0**	-	**0**	**0**	**0**	**3**
Cx. (Mcx.) aureus	**9**	**0**	**0**	**-**	**0**	-	**0**	**0**	**0**	**9**
Cx. (Mcx.) daumasturus	0	6	0	-	0	-	0	0	0	6
Cx. (Mcx.) dubitans	0	38	6	-	0	-	0	0	0	44
Cx. (Mcx.) fuscatus	0	21	1	-	0	-	0	0	0	22
Cx. (Mcx.) imitator	0	180	31	-	0	-	0	0	0	211
Cx. (Mcx.) inimitabilis	0	5	2	-	0	-	0	0	0	7
Cx. (Mcx.) lanei	0	2	0	-	0	-	0	0	0	2

Cx. (Mcx.) neglectus	0	45	5	-	0	-	0	0	0	**50**
Cx. (Mcx.) pleuristriatus	85	144	180	-	0	-	8	0	0	417
Cx. (Mcx.) reducens	0	0	0		0	-	0	0	0	0
Cx. (Mcx.) worontzowi	0	57	0	-	0	-	0	0	0	57
Cx. (Mel.) akritos	**1**	**0**	**0**	**-**	**0**	-	**0**	**0**	**0**	**1**
Cx. (Mel.) bahiensis	0	0	0	-	2	-	0	0	6	8
Cx. (Mel.) intrincatus	0	0	0	-	2	-	0	0	14	16
Cx. (Mel.) pilosus	**1**	0	0	**-**	**14**	-	**0**	**0**	**20**	**35**
Cx. (Mel.) ribeirensis	**0**	**0**	**0**	**-**	**8**	-	**0**	**0**	**0**	**8**
Cx. (Mel.) vaxus	0	0	0	-	89	-	0	0	0	89
Cx. ocellatus	0	13	0	-	0	-	0	0	0	13
Hg. (Con.) leucocelaenus	0	0	0	-	0	-	0	0	0	0
Li. durhami	611	0	0	-	0	-	0	0	0	611
Lu. (Lut.) bigoti	6	0	0	-	0	-	0	25	0	31
Ma. (Man.) indubitans	**0**	**0**	**0**	**-**	**3**	-	**0**	**0**	**0**	**3**
Ps. (Jan.) albigenu	**0**	**0**	**0**	**-**	**0**	-	**0**	**0**	**2**	**2**
Ps. (Jan.) ferox	0	0	0	-	3	-	0	0	11	14
Ru. (Run.) cerqueirai	0	0	0	-	0	-	0	0	0	0
Sa. (Sab.) purpureus	2	0	0	-	0	-	5	0	0	7
Sh. fluviatilis	0	0	0	-	0	-	0	0	0	0
Tr. pallidiventer	0	0	0	-	0	-	0	0	0	0
Tx. (Lyn.) portoricensis	**1**	**0**	**0**	**-**	**0**	-	**0**	**0**	**0**	**1**
Tx. (Lyn.) theobaldi	25	0	0	-	1	-	1	0	0	27
Tx. tricophygus	0	0	0	-	0	-	0	0	0	0
Ur. (Ura.) geometrica	0	0	0	-	24	-	0	0	0	24
Ur. (Ura.) lowii	0	0	0	-	5	-	0	0	0	5
Ur. (Ura.) pulcherrima	**0**	**0**	**0**	**-**	**3**	-	**0**	**0**	**0**	**3**
Wy. (Mim.) oblita	0	0	0	-	0	-	0	0	0	0
Wy. (Pho.) davisi	1	122	18	-	0	-	0	0	0	141

Wy. (Pho.) edwardsi	0	7	2	-	0	-	0	0	0	9
Wy. (Pho.) incaudata	**0**	**42**	**1**	**-**	**0**	-	**0**	**0**	**0**	**43**
Wy. (Pho.) pallidoventer	1	14	0	-	0	-	0	0	0	15
Wy. (Pho.) palmata	0	12	0	-	0	-	0	0	0	12
Wy. (Pho.) pilicauda	**0**	**14**	**1**	**-**	**0**	-	**0**	**0**	**0**	**15**
Wy. (Pho.) splendida	**0**	**4**	**0**	**-**	**0**	-	**0**	**0**	**0**	**0**
Wy. (Pho.) theobaldi	0	138	4	-	0	-	0	0	0	142
Wy. (Spi.) airosai	0	0	0	-	0	-	0	0	0	0
Wy. (Wyo.) lutzi	0	0	0	-	0	-	0	0	0	0
Wy. aporonoma	**0**	**0**	**0**	**-**	**0**	-	**7**	**0**	**0**	**7**
Wy. personata	0	0	0	-	0	-	0	0	0	0
Wy. serratoria	**0**	**1**	**0**	**-**	**0**	-	**0**	**0**	**0**	**1**
Total	1849	1068	304	0	515	0	99	302	338	4471

Table S2. Range of water volume, range of physicochemical parameters and coefficient of variation of different aquatic habitats explored during immature mosquito collections in two remnants of Atlantic Forest in the city of São Paulo, Brazil.

Area	Aquatic habitat	Volume (mL)	pH	Conductivity (µS/cm)	Total dissolved solids	Salinity (psu)
PEC	Artificial (N=30)	150 - 6000 CV:177%	4.52 - 8.44 CV:19%	30 - 639 CV:198%	15 - 319 CV:196%	0.01 - 0.31 CV:213%
	Epiphytic bromeliad (N=54)	100 - 1400 CV:221%	3.70 - 8.42 CV:19%	10 - 288 CV: 266%	5 - 144 CV:266%	0.01 - 0.14 CV:289%
	Ground bromeliad (N=2)	200 - 450 CV:54%	5.60 - 8.86 CV:20%	11 - 192 CV:204%	05 - 96 CV:202%	0.01 - 0.09 CV:218%
	Bamboo hole (N=4)	300 - 450 CV:29%	4.56 - 6.73 CV:20%	60 - 557 CV:268%	30 - 279 CV:268%	0.03 - 0.27 CV:290%
	Lake (N=11)	2000 - 12000 CV:138%	5.84 - 8.28 CV:19%	24 - 77 CV:203%	12 - 38 CV:201%	0.01 - 0.04 CV:212%
	Tree hollow (N=9)	100 - 500 CV:42%	5.80 - 7.69 CV:19%	27 - 1560 CV:199%	13 - 780 CV:197%	0.01 - 0.78 CV:212%
	Bamboo hollow (N=5)	200 - 300 CV:32%	5.21 - 6.89 CV:18%	188 - 2633 CV:203%	94 - 1316 CV:201%	0.09 - 1.37 CV:216%
	Pond (N=9)	800 - 16000 CV:240%	4.60 – 6.82 CV:19%	45 – 265 CV:266%	23 – 132 CV:266%	0.02 - 0.12 CV:289%
APA	Artificial (N=26)	150 - 10000 CV:152%	3.84 - 10.45 CV:19%	33 – 552 CV:258%	16 – 276 CV:258%	0.01 - 0.27 CV:281%
	Epiphytic bromeliad (N=62)	100 – 1000 CV:177%	3.57 – 5.85 CV:19%	12 – 749 CV:261%	6 – 375 CV:261%	0.00 - 0.36 CV:284%
	Ground bromeliad (N=9)	200 - 600 CV:199%	4.15 – 6.02 CV:19%	25 – 357 CV:266%	12 – 178 CV:266%	0.01 - 0.17 CV:289%
	Lake (N=22)	1200 - 16000 CV:160%	6.1 – 7.62 CV:19%	51 – 569 CV:265%	26 – 284 CV:265%	0.02 - 0.28 CV:289%
	Bamboo hollow (N=3)	350 - 500 CV:200%	6.50 - 7.89 CV:19%	499 – 6147 CV:262%	250 – 3074 CV:262%	0.24 – 3.31 CV:283%
	Rock pond (N=4)	1200 - 6000 CV:182%	6.18 - 6.44 CV:18%	35 – 224 CV:166%	17 – 113 CV:166%	0.01 - 0.11 CV:176%
	Pond (N=6)	3000 - 12000 CV:193%	5.99 – 7.49 CV:19%	38 – 380 CV:255%	19 – 190 CV:255%	0.02 - 0.18 CV:278%

CV = Coefficient of variation.
